# Refractory pain following hip arthroscopy: evaluation and management

**DOI:** 10.1093/jhps/hnx047

**Published:** 2018-01-18

**Authors:** Jason J Shin, Darren L de SA, Jeremy M Burnham, Craig S Mauro

**Affiliations:** Department of Orthopaedics and Sports Medicine, University of Pittsburgh Medical Center, Pittsburgh, PA 15203, USA

## Abstract

With increased knowledge and understanding of hip pathology, hip arthroscopy is rapidly becoming a popular treatment option for young patients with hip pain. Despite improved clinical and radiographic outcomes with arthroscopic treatment, some patients may have ongoing pain and less than satisfactory outcomes. While the reasons leading to failed hip arthroscopy are multifactorial, patient selection, surgical technique and rehabilitation all play a role. Patients with failed hip arthroscopy should undergo a thorough history and physical examination, as well as indicated imaging. A treatment plan should then be developed based on pertinent findings from the workup and in conjunction with the patient. Depending on the etiology of failed hip arthroscopy, management may be nonsurgical or surgical, which may include revision arthroscopic or open surgery, periacetabular osteotomy or joint arthroplasty. Revision surgery may be appropriate in settings including, but not limited to, incompletely treated femoroacetabular impingement, postoperative adhesions, heterotopic ossification, instability, hip dysplasia or advanced degeneration.

## INTRODUCTION

Major improvements in the understanding of hip pathomorphology, surgical implants and techniques, surgeon training and reliable arthroscopic procedures have resulted in increased popularity of arthroscopic hip preservation surgery. During the 5-year period from 2006 to 2010, the incidence of hip arthroscopy procedures performed by American Board of Orthopaedic Surgery Part II examinees increased by over 600% [1]. Such trends are also reflected in surgical training. Using publicly available Accreditation Council for Graduate Medical Education surgical case logs, Gil *et al.* [2] reported a 588% increase in hip arthroscopic procedures logged by residents between 2012 and 2013.

Hip arthroscopy has been used successfully as a treatment for both intra-articular [femoroacetabular impingement (FAI), labral tears, chondral lesions, loose bodies, synovial abnormalities, instability, septic arthritis] and extra-articular (snapping iliopsoas tendon/iliotibial band, gluteus tendon tears) conditions. Most published results of hip arthroscopy have reported favorable short and mid-term clinical and radiographic outcomes [3, 4]. However, with expanding indications for arthroscopic surgery and the subsequent increasing number of procedures being performed, the burden of refractory pain following hip arthroscopy will likely increase.

Failed hip arthroscopy may be defined as persistent postoperative pain and/or stiffness—diagnosed by a combination of decreased joint capacity and global range of motion (ROM)—that does not improve with nonsurgical means [5]. In a systematic review of 6134 patients, Harris *et al.* [6] reported a reoperation rate of 6.3% after index hip arthroscopy. The mean time to reoperation was 16.4 months with a conversion rate of 2.9% to total hip arthroplasty (THA). A more recent article analyzing indications and outcomes of only revision hip arthroscopy reported that the most common indications for revision hip arthroscopy were unaddressed or inadequately addressed FAI, labral tears and chondral lesions at the time of index surgery [4]. Additionally, the review reported that patients who most commonly underwent revision hip arthroscopy were female, younger (mean age of 33.4 years) and had a mean interval between index and revision hip arthroscopy of 25.6 months.

Management of refractory pain after hip arthroscopy is challenging, and reports on treatment options and outcomes are limited with small numbers. To appropriately manage these complex patients with refractory pain and optimize their outcomes, the surgeon must have a thorough understanding of various hip pathomorphologies and must be able to couple that knowledge with proper evaluation, workup and nonsurgical and surgical interventions.

## INITIAL WORKUP

### History and physical examination

Definitive diagnosis of the etiology of pain after hip arthroscopy may be multifactorial and not always obvious. Therefore, careful assessment to determine and confirm the underlying pathology is of utmost importance. Evaluation starts with thorough history, defining the location, duration and onset of pain and dysfunction. In order to determine if the symptoms are the result of the development of new pathology versus failure to adequately treat the initial pathology, the clinician must discern if the symptoms are new, different or continued following the index surgery. Moreover, patients should be inquired about activity level and any temporary resolution of symptoms postoperatively which may have returned at a later date. Previous medical records including studies performed prior to the index procedure as well operative notes, intraoperative images and implant sheets should be reviewed. Communication with the physical therapist to assess the patient’s participation and compliance with the postoperative course of physical therapy may also be useful. Although exceedingly rare in hip arthroscopy, when there is clinical suspicion of infection, appropriate tests should be collected, including inflammatory markers, cultures and sensitivities, to guide treatment with antibiotics and/or surgery. History and physical examination should also be focused on evaluating the patient to rule out disorders that may mimic the current symptoms.

Many potential causes of hip pain have overlapping physical examination findings, making accurate diagnosis even more challenging. Clinical examination should follow basic principles and include evaluation of the surgical incisions, alignment of the spine, pelvis and lower extremities, muscle atrophy and gait. Clinicians should pay particular attention to ROM deficits and weakness. Strength testing is important, and subtle muscular imbalance between the strong thigh muscles and relatively weak abdominal muscles may suggest athletic pubalgia symptoms. ROM of the back, hips and knees as well as generalized ligamentous laxity and hip stability must be assessed. Palpation of the pelvic structures may reveal conditions that would not benefit from hip arthroscopy such as sacroiliac joint pain. Neurologic examination should focus on the spine and look for signs of lumbar radiculopathy which can mimic thigh or groin pain. Physical examination alone is often insufficient for diagnosing the etiology of failed hip arthroscopy. Image-guided (either ultrasound or fluoroscopic) intra-articular hip injection with local anesthetic and/or a corticosteroid can be both diagnostic for distinguishing intra-/extra-articular from spinal pathology and therapeutic—though limited evidence exists in support of routine therapeutic use [7]. Especially following a failed hip arthroscopic procedure, an injection is a powerful test that can assist the surgeon in determining the presence of intra-articular hip lesions as the cause of symptom and disability. After the injection, patients are assessed for pain relief as they perform activities that normally precipitate symptoms [8]. If the injection does not result in at least temporary partial symptom relief, there may be other contributing pain generators and treatment of the hip may be less successful. Byrd and Jones reported 90% accuracy for detecting the presence of intra-articular abnormalities using intra-articular injection of anesthetic, and as such, a negative finding (e.g. no pain relief) from an intra-articular injection has a greater influence on decision-making in the revision setting against re-operation [9, 10]. A reasonable injection protocol would include a diagnostic/therapeutic hyaluronic acid (HA) intra-articular injection as first line. If a patient does not improve, and is still believed to be intra-articular, the next step should include a corticosteroid injection with radiological confirmation of intra-articular presence. This stems from the randomized, double-blind, cross-over study by Lee and colleagues [11]. In this study of 30 patients with clinical and radiographic evidence of FAI undergoing hip injection with either corticosteroid or HA, greater, statistically significant, improvements in hip disability and numeric pain ratings were observed in patients who first underwent an HA injection, with a corticosteroid injection 2 weeks later if no response to the initial HA injection. Additionally, selective injections to the extra-articular structures such as iliopsoas and trochanteric bursae may help delineate the source of pathology. There is limited evidence to suggest optimal dose, volume or frequency of intra-articular injections in the revision hip arthroscopic population.

### Imaging

**Fig. 1.**
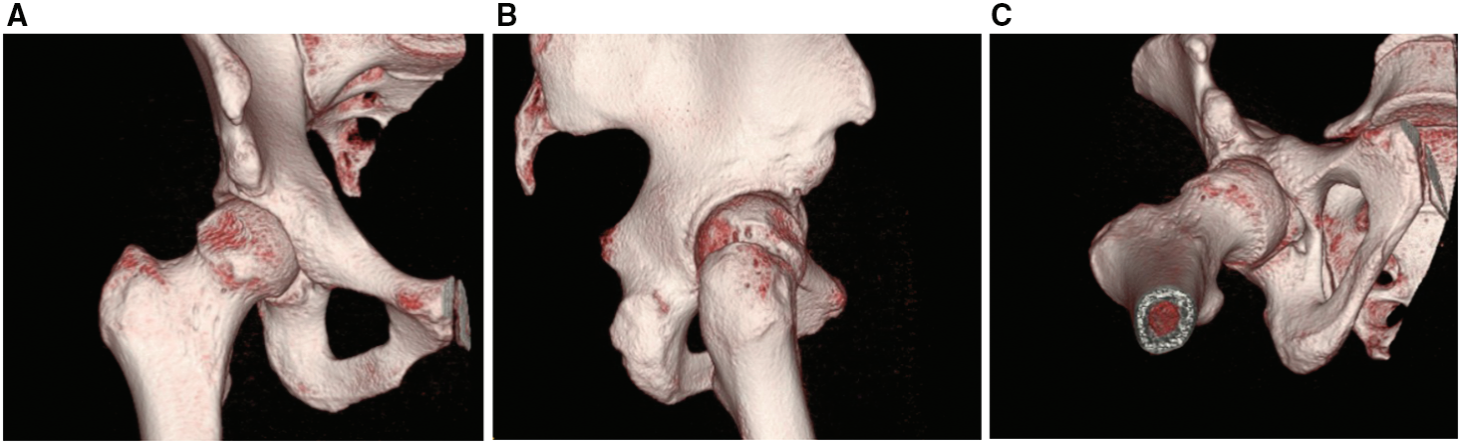
Coronal (**A**), sagittal (**B**) and axial (**C**) view of 3-D CT hip demonstrating post-surgical changes (overly aggressive anterior resection) and inadequately addressed residual cam where the deformity is persistent laterally and distally. Additionally, this patient demonstrates borderline acetabular dysplasia. The 3-D CT can provide invaluable information for preoperative planning.

Although not routinely acquired for index procedures, computerized tomography (CT) scan of the hip may be useful for both diagnostic and therapeutic planning, especially when revision arthroscopic or open surgery is being considered. Patients with FAI have a complex three-dimensional deformity of the hip joint. CT scans with three-dimensional reconstructions provide useful information of the bony morphology and is considered the gold standard for assessment of FAI [12]. CT scans are most helpful for determining the location and amount of resection that the surgeon needs to achieve for adequate correction, which may not be adequately assessed on plain films or cross-sectional MRI. In the revision setting, three-dimensional reconstruction is invaluable to determine the extent of osseous abnormalities and to critically assess the inadequate or excessive recontouring that was performed during initial surgery (Fig. 1). There is ongoing research in developing simulation software using 3-D CT for preoperative planning and prediction of postoperative ROM for patients undergoing hip preservation surgery [13–15]. With improved software and automation, such preoperative planning technology may gain popularity.

Even in light of imaging studies indicating residual bony pathology, MRI should be part of a thorough preoperative investigation, especially in the revision setting, to accurately evaluate the soft-tissue and cartilage of the hip. Although MRI may be adequate to assess chondral surfaces, effusions and diagnose avascular necrosis, use of intra-articular contrast improves sensitivity and specificity and is especially useful in evaluating for postoperative adhesions, capsule volume, capsular defects and recurrent labral tears. Using arthroscopy as the definitive diagnosis, Bryd and Jones compared the reliability of MRI with magnetic resonance arthrogram (MRA) and found improved sensitivity at detecting various lesions and a rate of false-negative results from 42% with MRI to 8% with MRA [9]. Mindful of resource allocation, a reasonable approach in light of current evidence would be to obtain an MRI with and without contrast to first confirm the presence and degree of effusion, and only if insufficient, proceed with an MRA.

In another study looking at 70 patients with recurrent symptoms after previous hip arthroscopy, McCarthy and Glassner assessed the association between MRA findings and intraoperative findings [16]. In patients with prior hip arthroscopy, the sensitivity, specificity, positive and negative predictive values of MRA was 82%, 70%, 94% and 39%, respectively, for diagnosis of labral disorders. The study’s reliability and accuracy for detecting labral disorders before revision arthroscopy are comparable with what has been reported for primary hip arthroscopy. The authors also noted that while MRA is highly accurate in detecting anterior labral tears, the anteromedial lesions were rarely diagnosed on MRA. For detecting loose bodies and chondral lesions, the authored reported that MRA is better at ruling in than in detecting such disorders. At the time of hip arthroscopy, anterior loose bodies were identified correctly, but only 2 of 10 of foveal loose bodies were identified correctly. Moreover, lower grade lesions (Grades I and II) were most difficult to detect and missed in 52% of cases on MRA. These findings emphasize the importance of combining imaging with patient demographics, thorough history and the surgeon’s physical examination to make the accurate diagnosis.

### Preoperative mental health and patient expectations

Several recent studies have suggested that preoperative mental health may play an important role in outcomes after orthopedic procedures. Wylie *et al.* quantified preoperative mental health in 169 patients with rotator cuff tears using the mental component portion of the Short Form 36 (SF36 MCS). The SF36 is a patient-reported outcome questionnaire that uses a variety of questions to quantify overall health status. The authors reported that shoulder function and symptoms correlated more with the patients’ mental health (as quantified by the SF36 MCS) than with tear size [17]. In a similar study, Jacobs *et al.* assessed 72 patients with intra-articular hip pathology. The authors quantified preoperative mental health using the mental component score from the Veteran’s Rand Survey (VR-12), which is comparable with the SF36. They found that mental health correlated more with preoperative symptomology than labral tear size or chondral lesion size as visualized during arthroscopy. Furthermore, patients with lower VR-12 mental component scores were less likely to return to work than patients with high VR-12 mental component scores [18]. These studies provide limited insight relative to the cause of low mental health scores in orthopedic patients, and the relationship between mental health and postoperative outcomes is not fully understood. Furthermore, it is unknown whether preoperative psychological intervention may affect outcomes in this patient population. Although the study of mental health relative to orthopedic outcomes is in its infancy, it would be prudent to assess and optimize the mental health of any patient with subpar surgical outcomes that are not definitively linked to an underlying structural problem.

The importance of identifying and discussing patient expectations both at index and prior to any intended revision procedure has not gone unnoticed. Patient expectations as it pertains to pain relief, mobility and return to sports/activities are often overly optimistic [19]. Given that, in general, postoperative outcomes are intimately related with fulfilling patient expectations, increasing efforts are currently underway, aimed at better capturing expectations to provide a realistic framework for patients [20]. One must be honest and upfront with expected rates of failure, rates of return to activities (particularly to desired preoperative levels) and the like.

Careful history and physical examination, appropriate imaging and diagnostic/therapeutic injections, and a holistic evaluation of failed hip arthroscopy patients can lead to accurate diagnosis and suitable therapy.

## MANAGEMENT OF FAILED HIP ARTHROSCOPY

**Fig. 2.**
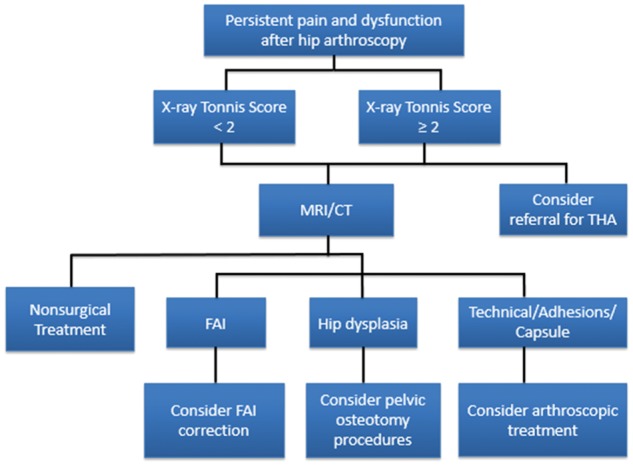
Treatment algorithm for failed hip arthroscopy.

As hip arthroscopy is a relatively emerging field, the literature regarding management and outcomes of failed hip arthroscopy is relatively sparse. However, in general, failed hip arthroscopy can be broadly categorized into one or more of the following: misdiagnosis, inadequate surgical procedure, poor healing, failure to treat concomitant pathology, inadequate or incomplete rehabilitation and/or a new injury or a complication (Table I). Moreover, compared with the presence of labral tears and/or mild chondral pathology alone, the presence of advanced arthritis has been associated with inferior outcomes [16]. The first step in management of the patient with a failed hip arthroscopy is to synthesize the history, physical examination, imaging studies and response to injections to come to understanding of the etiology of the failure. Prior to proceeding with operative management, it is prudent to look at the patient’s surgical reserve, their ability to partake in postoperative rehabilitation and rule out possibility of secondary gain. Regardless of management option, the surgeon should convey realistic expectations to the patient (Fig. 2).

**Table I.** Causes of failure

Incorrect diagnosis
Wrong part of hip addressed
Peritrochanteric disease
Psoas
Ligamentum teres
Instability
Spine
Core
Neurological
Vascular
Genitourinary
Gynecologic
Inadequate treatment
Persistent structural disease
Residual FAI
Dysplasia
Degenerative joint disease
Labral tear
Loose bodies
Inadequate rehabilitation or time
Poor healing
Complication
Postoperative adhesions
Heterotopic ossification
Technical failures
Dislodged anchor
Iatrogenic chondral damage
Overresection of labrum/acetabulum/femur
Capsular defect
Recurrent injury

## NONSURGICAL MANAGEMENT

In certain cases, a trial of physical therapy tailored to the individual needs of the patient may be indicated. A structured program comprised manual techniques, home exercises, gym program and graduated return to sport and physical activity may be beneficial. The deep hip rotator muscles including quadratus femoris, the gemelli and obturator internus act as the ‘rotator cuff of the hip joint’ and contribute to dynamic hip stability to steady the femoral head in the acetabulum [21]. Retraining and strengthening these groups may accelerate rehabilitation from the index procedure [22]. Stretching of both anterior and posterior capsule should be emphasized especially after labral surgery. Finally, the physical therapist will provide guidance to patients regarding graduated return to sports by introducing functional and sport-specific drills [22].

Communication with the physical therapist is invaluable in these situations to assess patient’s progress or lack of response to therapy. If the patient does not experience improvement or relief of symptoms with nonsurgical treatments, depending on the etiology, surgical management of failed hip arthroscopy includes one of the following options: revision hip arthroscopy, revision open hip preservation with or without osteotomy or THA.

Although the role of biologics and platelet-rich plasma (PRP) injections specifically in the setting of failed hip arthroscopy has not been studied in detail, there is some limited literature supporting its use in early arthritis. A recent RCT reported that compared with HA, PRP injections resulted in improved pain scores and patient reported outcomes at 6 months. Moreover, no complications were observed from the injections [23]. However, the mechanism by which PRP exerts its role has not yet been cleared defined and is part of ongoing investigation. Given the potential to reduce pain and improve function and quality of life, PRP may be used as an adjunct.

## RESIDUAL IMPINGEMENT

**Fig. 3.**
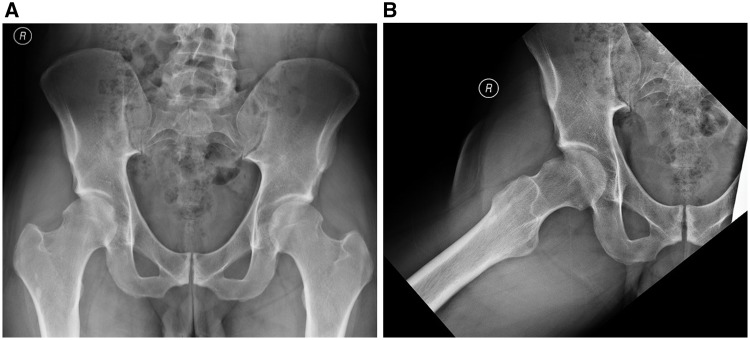
Anteroposterior pelvis (**A**) and Dunn (**B**) views of a patient (same as in Fig. 1) with persistent right hip pain that presented to clinic for second opinion after index arthroscopic surgery. There is evidence of inadequately addressed impingement (e.g. residual cam distally and laterally; over-resection anteriorly) and borderline dysplasia.

Numerous studies have identified unaddressed or inadequately addressed FAI at index surgery as the leading indication for revision hip preservation surgery [6, 24–27]. In the largest series to date on revision hip arthroscopy patients with residual FAI treated by a single surgeon, the vast majority of cases (>90%) had residual cam-type femoral morphology with inadequate head–neck offset and residual asphericity, which was located most commonly at the superoposterior/lateral head–neck junction (Fig. 3). Residual pincer-type disease that required surgical treatment was also noted in 70.3% of cases, including, residual cranial retroversion, prominent/low anterior inferior iliac spine (AIIS)/subspine impingement or profunda deformity [26]. Most commonly, combined cam- and pincer type FAI was noted, followed by isolated cam-type. Isolated pincer-type variants were infrequently noted in their series of revision cases [26]. It is worth noting that in this series of patients who underwent revision arthroscopic surgery by a single high volume hip arthroscopist, all patients underwent 3-D CT imaging for preoperative evaluation of the architectural abnormalities. CT scans are often necessary in the revision setting to properly classify the cam lesion’s location and size. Ross *et al.* [28] correlated preoperative 3-D CT with specific intraoperative fluoroscopic image to characterize the cam deformity. The authors describe six consistent intraoperative fluoroscopy views: three views in hip extension and three views in hip flexion of 50 degrees, which correlate with CT scans and are helpful in localization and visualization of the typical cam deformity [28]. A comprehensive understanding of the FAI morphology will result in a more precise resection.

During revision resection, medial cam deformities can be accessed by introducing a burr through the midanterior portal with the hip in flexion, external rotation and abduction, while laterally based cam deformities can be accessed with the burr in anterolateral or posterolateral portal with the hip positioned in extension, internal rotation and adduction [3]. As in all cases of femoroplasty, the surgeon must be aware of their anatomical position in space and protect the medial and lateral retinacular vessels which supply the femoral head [3].

Residual FAI is a positive predictive factor for success of revision hip arthroscopy. Achieving greater postoperative head–neck offset and subspine/AIIS decompression are significant predictors for better patient-reported outcome measures [26]. In a cohort study comparing patients who underwent revision arthroscopic FAI correction with those who underwent primary arthroscopic FAI correction, Larson *et al.* [26] reported significant improvements in both groups as measured by modified Harris Hip Score (mHHS), Short Form–12 and pain on a visual analog scale. However, the outcomes measures after revision surgery were inferior to those after primary arthroscopic FAI corrective surgery on each of the three outcome measures. Good/excellent results were noted in 81.7% of primary arthroscopic FAI correction compared with 62.7% good/excellent results in revision surgery. Despite the reduced magnitude of clinical improvement in the revision cohort, the association between residual FAI and overall improved outcome in revision hip arthroscopy is likely related to identifying a specific pathomorphologic anatomy that can reliably be addressed with revision surgery [29].

## LABRAL TEAR

**Fig. 4.**
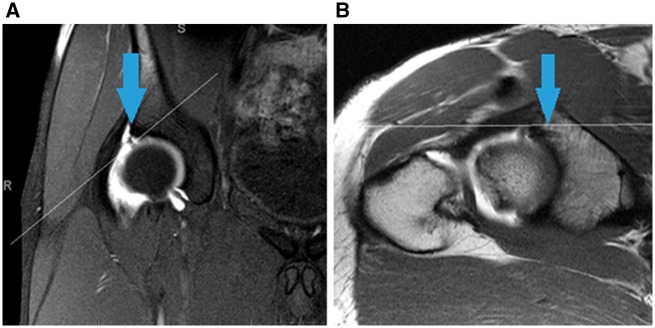
T1 coronal (**A**) and oblique axial (**B**) MRA of the hip demonstrating a labral tear at the 1 o’clock position (A, blue arrow) and a suture anchor from prior surgery (B, blue arrow).

Recent studies have demonstrated that labral tears rarely occur in the absence of bony abnormalities [30, 31]. Given that residual impingement is the most frequently cited reason for revision hip arthroscopy, it comes as no surprise that tears in the labrum are often observed at the time of repeat surgery (Fig. 4). One study reported observing a labrum tear in 86% of hips at revision arthroscopy [16]. Unless the underlying bony morphology is adequately addressed, even a well-repaired labrum from the time of index surgery is unlikely to be able to withstand the repeat exposure to repetitive forces which caused the original labral injury. This makes treatment of bony abnormalities essential to the long-term success of the treatment of soft tissue lesions [30].

Labrum preservation surgery has been reported to be a significant predictor for better outcomes after revision surgery [26]. In recent study looking at 85 hips that underwent arthroscopic revision FAI corrective surgery, labrum was debrided in 27 hips, repaired in 49 hips, reconstructed in 7 hips and stable in 2 hips. Larson *et al.* [26] found that labra which were repaired or reconstructed with tibialis anterior allograft tendon had significantly greater improvement in mHHS value compared with debrided labrum in arthroscopic revision surgery. Similar improvements were reported by Domb *et al.* [25] where labral reconstruction during revision surgery was associated with improved outcome. Such clinical outcomes reflect cadaveric studies which show improved hip biomechanics in intact labrum compared with labral deficient states.

When possible, the labrum is kept and repaired. The functional roles of acetabular labrum include shock absorption, joint lubrication, pressure distribution, maintenance of hip stability and suction-seal effect [3, 32]. Extrapolating results from studies comparing debridement to refixation, patients-reported lower outcome measure scores and less satisfaction following labrum resection. 

## HETEROTOPIC OSSIFICATION

Heterotopic ossification (HO) is abnormal bone formation in soft tissue where bone normally does not exist. Under the stresses of postoperative tissue damage, the cellular environment promotes angiogenesis, fibroproliferation and endochondral ossification, leading to ectopic bone growth located anterior to the hip joint. With reported rates of up to 44%, HO is one of the most common complications after hip arthroscopy [33]. Although HO after hip arthroscopy is an asymptomatic incidental finding in most patients, it can also result in impaired function secondary to pain, impingement and decreased ROM [34]. As a result, the clinical presentation of HO after hip arthroscopy can often be difficult to isolate from other sources of postoperative stiffness and pain. Plain radiographs may demonstrate cloud like hyper-density which matures into solid bone by 3 months. Severity of ossification is best assessed with CT and 3-D reformats should be obtained when planning for revision surgery for removal of HO to visualize the shape and map the location of the ectopic bone [34].

The adage that ‘prevention is the best medicine’ is especially true for HO. Numerous retrospective studies as well as a recent double-blinded randomized placebo-controlled trial [35] have shown that various nonsteroidal anti-inflammatory drug (NSAID) prophylaxis significantly reduces the incidence of HO in patients undergoing hip arthroscopy [33, 35–37]. If however, the patient develops symptoms and dysfunction, and goes on to fail non-surgical treatment and rehabilitation, the patient may be a candidate for surgical excision of HO. Timing of intervention is critical and should be delayed until 6–12 months after the index surgery, when the radiographic appearance is consistent with dense cortical bone. This delay allows the HO to mature and has been shown to decrease the rate of recurrence. Bedi *et al.* [36] reported on seven cases HO that required excision (three arthroscopic revision procedures and four open resection). At the final follow-up, there were no cases of recurrence of HO after excision. To minimize recurrence after excision of HO, if there are no contraindications, the patients should be placed on prophylactic NSAIDs.

## ADHESIONS

**Fig. 5.**
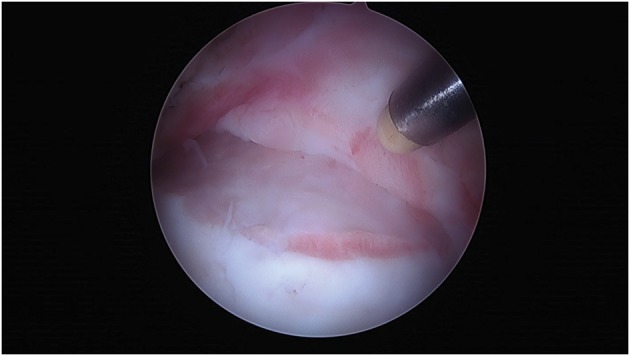
Adhesions often occur at the site of neck osteochondroplasty where there is good vascularity. When the adhesions form proximally in the area of the resected femoral neck, they can restrict motion and become symptomatic.

Intra-articular hip adhesions are a significant source of failure and pain following arthroscopic surgery for FAI. Most adhesions occur at the site of neck osteochondroplasty or between site of labral repair and the capsule where there is good vascularity (Fig. 5) [30]. As part of the normal healing process, adhesions are caused by scar tissue. It is when the adhesions form more proximal in the area of the resected femoral neck, thus restricting motion, that they become symptomatic. In flexion, adhesions can impinge against the acetabulum rim, which may lead to labral re-rupture. Adhesions can also slide into the joint, causing cartilaginous and osseous damage. In a review of 1264 patients with at least 1-year follow-up, Willimon *et al.* [38] identified 57 patients that underwent repeat arthroscopies. All of the 57 revision cases had adhesions at the time of repeat arthroscopy [38]. In general, patients who develop adhesions present with groin pain and limitation of motion, and tend to be younger and report lower patient-reported outcomes. In one study, patients under 30 years of age were 5.9 times more likely to have adhesions while patients with mHHS scores less than 50 were 2.4 times more likely [38]. In addition to the use of prophylactic NSAIDS, adhesions can be reduced with early postoperative ROM and circumduction exercises.

After excluding other causes of symptoms, patients with suspected adhesions should undergo advanced imaging. Intra-articular adhesions are best visualized using MRA on coronal and sagittal images [39, 40]. With symptoms that are recalcitrant to physical therapy, hip arthroscopy with resection of adhesion is indicated. Arthroscopic release of the adhesions has been shown to improve outcomes even after previous open surgical hip dislocation [41]. In the setting of symptomatic adhesions, the objective of revision hip arthroscopy is to release and remove labral and capsular adhesions and to smooth the surface of the labrum. A radiofrequency probe and a shaver can be used to free the labrum off the capsule and to open the joint space down to the capsular attachment by the superolateral base of the femoral neck. Adhesions are usually not found in the medial part of the joint. After adhesiolysis, the hip joint should be checked for impingement-free ROM and postoperatively an aggressive motion program is implemented for prophylaxis against recurrent adhesions [39].

## HIP INSTABILITY

The stability of the hip is multifactorial and is determined by a balance between osseous and soft-tissue restraints. There is an increasing body of evidence on the interplay between arthroscopy, capsular management and postoperative instability with hip arthroscopists generally accepting that capsular and labral management, psoas tenotomy and acetabuloplasty play a role in iatrogenic hip instability [42].

Most postsurgical dislocations occur anteriorly with the hip positioned in extension and external rotation [43, 44]. The iliofemoral ligament and the capsule are important structures in maintaining hip stability [45]. In hip arthroscopy, when the iliofemoral ligament is cut during interportal capsulotomy, the capsule loses its ability to resist external rotation and extension of the hip. In fact, capsulotomy has been shown to result in increased motion and altered biomechanics in cadaveric models [46, 47]. Thus, if the interportal capsulotomy is left unrepaired, the hip may be predisposed to micro- or macroinstability. This instability created with an unrepaired capsulotomy may be amplified in cases where a psoas release is performed [48].

Although, patients who present with frank dislocation are easily identified for revision surgery, patients with microinstability may present with ill-defined symptoms. These patients may complain of mechanical symptoms such as clicking, locking and catching. They may experience a sense of giving way and pain with hip external rotation and extension. Specific physical examination findings in the unstable hip include apprehension when hip is placed in traction as well as apprehension with posterior impingement test and a positive dial test. Like with all other joints, it is important to check the contralateral hip to assess side-to-side difference. Imaging, including MRA, can be used as adjuncts to evaluate for patulous or redundant capsule and to identify the capsular defects from inadequate healing.

Symptomatic patients with iatrogenic hip instability may be candidates for revision hip arthroscopy. During revision surgery, the edges of the previous capsulotomy are often covered in scar tissue and may be difficult to identify. In these cases, the scar tissue can be debrided with a shaver so that the native capsular edges can be identified and then repaired by passing stitches using various suture-passing instruments to obtain a tight closure [49]. In setting of previous capsulectomy, where adequate native tissue for a repair is unavailable, some described techniques include capsular reconstruction with an iliotibial band, rectus femoris or acellular dermal matrix were used [49–51]. On the other spectrum, where there is excessive or redundant capsular tissue, a plication may be more appropriate than simple capsular closure. The technique previously described by Domb *et al.* consists of imbricating and shifting the capsule inferiorly by taking larger bites of the capsule on the distal side [52]. Postoperatively, patients are instructed to avoid combined hip extension and external rotation for 6 weeks.

In revision settings, capsular repair has been reported to be an independent predictor of improved outcomes [26]. In a study of 33 patients who developed symptomatic instability requiring a revision surgery for capsulorrhaphy, at the final follow-up of minimum 12 months, the authors reported significant improvements in patient-reported outcome scores [49]. In this series, no recurrent hip subluxation or dislocations were reported after the repair.

## OTHER PATHOLOGY

There exists a separate clinical population (e.g. those with synovial chondromatosis, pigmented villonodullar synovitis (PVNS) or systematic/rheumatoid conditions) who may fail index hip arthroscopic management. The recurrence rate for synovial chondromatosis after arthroscopic management is 7.1% [53]. Arthroscopy is particularly beneficial for diagnosis and management of lesions <10 mm [54], and revision surgery may not be necessary for residual loose bodies. Though specific criteria for revision surgery in this population is lacking, factors to consider prior to revision surgery include, but are not limited to, number and size of loose bodies, location (i.e. peripheral versus central compartment), degree of pain, presence of mechanical symptoms and failure of conservative treatment. With regards to failed hip arthroscopy for PVNS or systemic/rheumatoid conditions, there is a paucity of data on the best approaches for both index and revision cases. Whether or not the revision procedure would be amenable to repeat arthroscopy hinges on severity of tissue burden and degree of underlying cartilage damage, unless effect may remain [55, 56].

## INAPPROPRIATE INDICATIONS: ARTHRITIS AND HIP DYSPLASIA

An association between advanced degenerative arthritis and inferior outcomes after hip arthroscopy is well documented [4, 57, 58]. A large meta-analysis of over 6000 hip arthroscopy patients by Harris *et al.* [6] found that conversion to THA was the most common reason for revision surgery. In a recent systematic review, Domb *et al.* [24] analyzed data from 15 articles which included 2051 hips that underwent hip arthroscopy. The authors reported no conversion to arthroplasty in patients with Tonnis Grade 0, whereas patients with Grade 2 or greater had 17.5% conversion rate. Based on their review, the authors recommended that arthroplasty should be considered in patients with Tonnis grade of 2 or greater or a joint space of 2 mm or less, rather than hip arthroscopy [24]. The surgeon must carefully analyte plain radiographs, including the false-profile view, to assess the anterior, posterior and inferior hip joint space narrowing that may be undetectable on frontal view [59].

Additionally, the treating surgeon should make every effort to obtain and review the intraoperative notes and images from the index surgery. Even in setting of preserved joint space, the presence of focal Grade 3 or 4 chondral lesions on MRI and/or arthroscopy are independent predictors for poorer patient-reported outcomes and higher failure rates [26, 60–62]. In a population-based study looking at 1577 patients, the authors reported that an age greater than 50 years and undergoing a chondroplasty of hip at the time of index procedure to be predictive of conversion to arthroplasty [57]. Interestingly, this study reported that over 90% who failed arthroscopic treatment had arthroplasty within 2 years, suggesting that when patients fail hip arthroscopy, conversion to THA occurs quickly [57]. As with any surgical procedure, the importance of proper patient selection in achieving favorable outcomes after hip arthroscopy cannot be underestimated. When dealing with patients with arthritic hips, it may be useful to provide counsel about the limited therapeutic effect of arthroscopy, the likelihood of eventual THA and, when indicated, offer arthroplasty [58].

An additional structural deformity that the surgeon must consider in failed arthroscopy is hip dysplasia. Acetabular dysplasia and FAI pathomorphology frequently coexist, which further complicate diagnostic and treatment decision. Patients may present with anterolateral pain and limp that is accentuated by abductor fatigue. Although it is difficult to precisely define acetabular dysplasia, generally it is a structural deformity consisting of a large spectrum of acetabulum retroversion with anterior overcoverage, posterior undercoverage, lateral center edge angle less than 20 degrees and increased inclination or Tonnis angle greater than 10 degrees.

Though select exceptions (e.g. dynamic impingement, micro instability) [63], most hip arthroscopy experts caution against the use of arthroscopic surgery in setting of acetabular dysplasia, especially with center-edge angles less than 20 degrees [64], while borderline dysplasia is also cautioned [3]. In a study of 58 patients who failed hip arthroscopy and were treated with either arthroscopic or open revision procedure, 24% hips of the hips were determined to have dysplasia and were subsequently treated with acetabular reorientation via the Bernese periacetabular osteotomy (PAO) [65]. The authors stated that in patients with symptomatic dysplasia, while repair of associated labral lesions is possible arthroscopically, the underlying structural deformity requires redirectional acetabular osteotomy. Citing compromised biomechanics of the hip and increasing stresses on the lateral region of the acetabulum, Parvizi *et al.* [66] reported poor outcomes with labral repair in patients with underlying hip dysplasia. In the case of hip dysplasia, deformity correction should be considered and the most optimal technique to address the lesion should be determined. Although details regarding open procedures are beyond the scope of this review article, where necessary, open procedures, such as PAO, should be recommended.

## TIMING

The trajectory of a patient’s recovery post-hip arthroscopy is widely variable and influenced by numerous factors encompassing patient characteristics, underlying diagnoses, procedures performed and specific postoperative rehabilitation protocols. In much the same way, the diagnostic workup and management strategy of a patient who has failed hip arthroscopy requires an individualized, stepwise approach. This is compounded by the fact that interpreting post-hip arthroscopic modalities (such as MRI) can prove quite difficult due to post-surgical changes. For example, Kim *et al.* compared the post-arthroscopic MRA findings in symptomatic and asymptomatic patients and found no differences in the presence of adhesions, residual labral tears and hip capsule defects [67]. Though no ideal framework exists in the literature regarding appropriate times for investigations, injections or revision surgery—be it arthroscopic or open—careful attention must be paid to fully characterizing the means of failure prior to embarking on surgical intervention.

## SUMMARY

Overall, outcomes of hip arthroscopy are favorable. With expanding indications for treatment as well as growing transfer of knowledge and skill, the number of arthroscopic hip procedures being performed will likely continue to increase. Likewise, the number of failures will also increase.

For optimal results, with careful consideration for symptom history and workup, the clinician must accurately diagnose the etiology of failure, which include unaddressed bony abnormalities and dysplasia, inadequately addressed FAI, labral and chondral defects, postsurgical stiffness and iatrogenic instability. Patients with refractory symptoms may be managed nonsurgically or surgically, depending on the etiology of the failure. Depending on the underlying diagnosis, patients can be managed surgically with either arthroscopic or open procedures—including osteotomies and arthroplasty. Although to date, published outcome studies are short- to mid-term, future long-term studies will help determine appropriate course of treatment.

## CONFLICT OF INTEREST STATEMENT

None declared.

## FUNDING

Authors have received no funding for this work.

## References

[1] Bozic KJ, Chan V, Valone FH Trends in hip arthroscopy utilization in the United States. J Arthroplasty 2013; 28: 140–3. 23916639 10.1016/j.arth.2013.02.039

[2] Gil JA, Waryasz GR, Owens BD Variability of arthroscopy case volume in orthopaedic surgery residency. Arthrosc J Arthrosc Relat Surg off Publ Arthrosc Assoc N Am Int Arthrosc Assoc 2016; 32: 892–7. 10.1016/j.arthro.2016.01.018 26993670

[3] Bedi A, Ross JR, Kelly BT Avoiding complications and treating failures of arthroscopic femoroacetabular impingement correction. Instr Course Lect 2015; 64: 297–306. 25745915

[4] Bryan AJ, Krych AJ, Pareek A Are short-term outcomes of hip arthroscopy in patients 55 years and older inferior to those in younger patients? Am J Sports Med 2016; 44: 2526–30. 27416992 10.1177/0363546516652114

[5] de Sa D, Phillips M, Catapano M Adhesive capsulitis of the hip: a review addressing diagnosis, treatment and outcomes. J Hip Preserv Surg 2016; 3: 43–55. 27026818 10.1093/jhps/hnv075 PMC4808257

[6] Harris JD, McCormick FM, Abrams GD Complications and reoperations during and after hip arthroscopy: a systematic review of 92 studies and more than 6, 000 patients. Arthrosc J Arthrosc Relat Surg off Publ Arthrosc Assoc N Am Int Arthrosc Assoc 2013; 29: 589–95. 10.1016/j.arthro.2012.11.003 23544691

[7] Chandrasekaran S, Lodhia P, Suarez-Ahedo C Symposium: evidence for the use of intra-articular cortisone or hyaluronic acid injection in the hip. J Hip Preserv Surg 2016; 3: 5–15. 27026814 10.1093/jhps/hnv020 PMC4808252

[8] Byrd JWT. Femoroacetabular impingement in athletes: current concepts. Am J Sports Med 2014; 42: 737–51. 23982400 10.1177/0363546513499136

[9] Byrd JWT, Jones KS. Diagnostic accuracy of clinical assessment, magnetic resonance imaging, magnetic resonance arthrography, and intra-articular injection in hip arthroscopy patients. Am J Sports Med 2004; 32: 1668–74. 15494331 10.1177/0363546504266480

[10] Khan W, Khan M, Alradwan H Utility of intra-articular hip injections for femoroacetabular impingement: a systematic review. Orthop J Sports Med 2015; 3: 2325967115601030. 26535395 10.1177/2325967115601030 PMC4622294

[11] Lee YK, Lee GY, Lee JW Intra-articular injections in patients with femoroacetabular impingement: a prospective, randomized, double-blind, cross-over study. J Korean Med Sci 2016; 31: 1822–7. 27709863 10.3346/jkms.2016.31.11.1822 PMC5056217

[12] Milone MT, Bedi A, Poultsides L Novel CT-based three-dimensional software improves the characterization of cam morphology. Clin Orthop 2013; 471: 2484–91. 23361933 10.1007/s11999-013-2809-x PMC3705074

[13] Audenaert EA, Mahieu P, Pattyn C. Three-dimensional assessment of cam engagement in femoroacetabular impingement. Arthrosc J Arthrosc Relat Surg off Publ Arthrosc Assoc N Am Int Arthrosc Assoc 2011; 27: 167–71. 10.1016/j.arthro.2010.06.031 20952150

[14] Kuhn AW, Ross JR, Bedi A. Three-dimensional imaging and computer navigation in planning for hip preservation surgery. Sports Med Arthrosc Rev 2015; 23: e31–8. 26524559 10.1097/JSA.0000000000000094

[15] Tannast M, Kubiak-Langer M, Langlotz F Noninvasive three-dimensional assessment of femoroacetabular impingement. J Orthop Res off Publ Orthop Res Soc 2007; 25: 122–31. 10.1002/jor.20309 17054112

[16] McCarthy JC, Glassner PJ. Correlation of magnetic resonance arthrography with revision hip arthroscopy. Clin Orthop 2013; 471: 4006–11. 23904247 10.1007/s11999-013-3202-5 PMC3825902

[17] Wylie JD, Suter T, Potter MQ Mental health has a stronger association with patient-reported shoulder pain and function than tear size in patients with full-thickness rotator cuff tears. J Bone Joint Surg Am 2016; 98: 251–6. 26888672 10.2106/JBJS.O.00444

[18] Jacobs C, Burnham J, King P Preoperative symptoms in femoroacetabular impingement patients are more related to mental health scores than the severity of labral tear or chondral lesion. *American Academy of Orthoapedic Surgeons 2017 Annual Meeting* San Diego, CA, 2017. 10.1016/j.arth.2017.06.053 28739309

[19] Mannion AF, Impellizzeri FM, Naal FD Fulfilment of patient-rated expectations predicts the outcome of surgery for femoroacetabular impingement. Osteoarthritis Cartilage 2013; 21: 44–50. 23069854 10.1016/j.joca.2012.09.013

[20] Mancuso CA, Wentzel CH, Ghomrawi HMK Hip preservation surgery expectations survey: a new method to measure patients’ preoperative expectations. Arthrosc J Arthrosc Relat Surg off Publ Arthrosc Assoc N Am Int Arthrosc Assoc 2017; 33: 959–68. 10.1016/j.arthro.2016.11.012 28049596

[21] Retchford TH, Crossley KM, Grimaldi A Can local muscles augment stability in the hip? A narrative literature review. J Musculoskelet Neuronal Interact 2013; 13: 1–12. 23445909

[22] Bennell KL, O’Donnell JM, Takla A Efficacy of a physiotherapy rehabilitation program for individuals undergoing arthroscopic management of femoroacetabular impingement—the FAIR trial: a randomised controlled trial protocol. BMC Musculoskelet Disord 2014; 15: 58. 24571824 10.1186/1471-2474-15-58 PMC3941691

[23] Dallari D, Stagni C, Rani N Ultrasound-guided injection of platelet-rich plasma and hyaluronic acid, separately and in combination, for hip osteoarthritis: a randomized controlled study. Am J Sports Med 2016; 44: 664–71. 26797697 10.1177/0363546515620383

[24] Domb BG, Gui C, Lodhia P. How much arthritis is too much for hip arthroscopy: a systematic review. Arthrosc J Arthrosc Relat Surg 2015; 31: 520–9. 10.1016/j.arthro.2014.11.008 25543247

[25] Domb BG, Stake CE, Lindner D Revision hip preservation surgery with hip arthroscopy: clinical outcomes. Arthrosc J Arthrosc Relat Surg off Publ Arthrosc Assoc N Am Int Arthrosc Assoc 2014; 30: 581–7. 10.1016/j.arthro.2014.02.005 24642106

[26] Larson CM, Giveans MR, Samuelson KM Arthroscopic hip revision surgery for residual femoroacetabular impingement (FAI) surgical outcomes compared with a matched cohort after primary arthroscopic FAI Correction. Am J Sports Med 2014; 42: 1785–90. 24875469 10.1177/0363546514534181

[27] Sardana V, Philippon MJ, de Sa D Revision hip arthroscopy indications and outcomes: a systematic review. Arthrosc J Arthrosc Relat Surg off Publ Arthrosc Assoc N Am Int Arthrosc Assoc 2015; 31: 2047–55. 10.1016/j.arthro.2015.03.039 26033461

[28] Ross JR, Bedi A, Stone RM Intraoperative fluoroscopic imaging to treat cam deformities correlation with 3-dimensional computed tomography. Am J Sports Med 2014; 42: 1370–6. 24737661 10.1177/0363546514529515

[29] Cvetanovich GL, Harris JD, Erickson BJ Revision hip arthroscopy: a systematic review of diagnoses, operative findings, and outcomes. Arthrosc J Arthrosc Relat Surg 2015; 31: 1382–90. 10.1016/j.arthro.2014.12.027 25703289

[30] Philippon MJ, Schenker ML, Briggs KK Revision hip arthroscopy. Am J Sports Med 2007; 35: 1918–21. 17703000 10.1177/0363546507305097

[31] Wenger DE, Kendell KR, Miner MR Acetabular labral tears rarely occur in the absence of bony abnormalities. Clin Orthop 2004; 426: 145–50. 10.1097/01.blo.0000136903.01368.20 15346066

[32] Ward JP, Rogers P, Youm T. Failed hip arthroscopy: causes and treatment options. Orthopedics 2012; 35: 612–7. 22784891 10.3928/01477447-20120621-11

[33] Rath E, Sherman H, Sampson TG The incidence of heterotopic ossification in hip arthroscopy. Arthrosc J Arthrosc Relat Surg off Publ Arthrosc Assoc N Am Int Arthrosc Assoc 2013; 29: 427–33. 10.1016/j.arthro.2012.10.015 23351728

[34] Amar E, Sharfman ZT, Rath E. Heterotopic ossification after hip arthroscopy. J Hip Preserv Surg 2015; 2: 355–63. 27011859 10.1093/jhps/hnv052 PMC4732379

[35] Beckmann JT, Wylie JD, Potter MQ Effect of naproxen prophylaxis on heterotopic ossification following hip arthroscopy: a double-blind randomized placebo-controlled trial. J Bone Joint Surg Am 2015; 97: 2032–7. 26677237 10.2106/JBJS.N.01156 PMC4673445

[36] Bedi A, Zbeda RM, Bueno VF The incidence of heterotopic ossification after hip arthroscopy. Am J Sports Med 2012; 40: 854–63. 22268230 10.1177/0363546511434285

[37] Randelli F, Pierannunzii L, Banci L Heterotopic ossifications after arthroscopic management of femoroacetabular impingement: the role of NSAID prophylaxis. J Orthop Traumatol off J Ital Soc Orthop Traumatol 2010; 11: 245–50. 10.1007/s10195-010-0121-z PMC3014465 21116673

[38] Willimon SC, Briggs KK, Philippon MJ. Intra-articular adhesions following hip arthroscopy: a risk factor analysis. Knee Surg Sports Traumatol Arthrosc 2014; 22: 822–5. 24162717 10.1007/s00167-013-2728-0

[39] Beck M. Groin Pain after Open FAI Surgery: the role of intraarticular adhesions. Clin Orthop 2009; 467: 769–74. 19082679 10.1007/s11999-008-0653-1 PMC2635436

[40] Crim J. Imaging evaluation of the hip after arthroscopic surgery for femoroacetabular impingement. Skeletal Radiol 2017; 46:1315–26. 28466104 10.1007/s00256-017-2665-y PMC5559574

[41] Krueger A, Leunig M, Siebenrock KA Hip arthroscopy after previous surgical hip dislocation for femoroacetabular impingement. Arthrosc J Arthrosc Relat Surg 2007; 23: 1285–9.e1. 10.1016/j.arthro.2007.07.002 18063171

[42] Ortiz-Declet V, Mu B, Chen AW Should the capsule be repaired or plicated after hip arthroscopy for labral tears associated with femoroacetabular impingement or instability? A systematic review. Arthrosc J Arthrosc Relat Surg off Publ Arthrosc Assoc N Am Int Arthrosc Assoc 2017; DOI: 10.1016/j.arthro.2017.06.030.

[43] Ranawat AS, McClincy M, Sekiya JK. Anterior dislocation of the hip after arthroscopy in a patient with capsular laxity of the hip. A case report. J Bone Joint Surg Am 2009; 91: 192–7. 19122095 10.2106/JBJS.G.01367

[44] Matsuda DK. Acute iatrogenic dislocation following hip impingement arthroscopic surgery. Arthrosc J Arthrosc Relat Surg off Publ Arthrosc Assoc N Am Int Arthrosc Assoc 2009; 25: 400–4. 10.1016/j.arthro.2008.12.011 19341927

[45] Telleria JJM, Lindsey DP, Giori NJ An anatomic arthroscopic description of the hip capsular ligaments for the hip arthroscopist. Arthrosc J Arthrosc Relat Surg off Publ Arthrosc Assoc N Am Int Arthrosc Assoc 2011; 27: 628–36. 10.1016/j.arthro.2011.01.007 21663720

[46] Abrams GD, Hart MA, Takami K Biomechanical evaluation of capsulotomy, capsulectomy, and capsular repair on hip rotation. Arthrosc J Arthrosc Relat Surg 2015; 31: 1511–7. 10.1016/j.arthro.2015.02.031 25882176

[47] Jackson TJ, Peterson AB, Akeda M Biomechanical effects of capsular shift in the treatment of hip microinstability creation and testing of a novel hip instability model. Am J Sports Med 2016; 44: 689–95. 26717973 10.1177/0363546515620391

[48] Yeung M, Memon M, Simunovic N Gross instability after hip arthroscopy: an analysis of case reports evaluating surgical and patient factors. Arthrosc J Arthrosc Relat Surg off Publ Arthrosc Assoc N Am Int Arthrosc Assoc 2016; 32: 1196–204.e1. 10.1016/j.arthro.2016.01.011 27013107

[49] Wylie JD, Beckmann JT, Maak TG Arthroscopic capsular repair for symptomatic hip instability after previous hip arthroscopic surgery. Am J Sports Med 2016; 44: 39–45. 26419897 10.1177/0363546515608162

[50] Dierckman BD, Guanche CA. Anterior hip capsuloligamentous reconstruction for recurrent instability after hip arthroscopy. Am J Orthop Belle Mead NJ 2014; 43: E319–23. 25490020

[51] Trindade CAC, Sawyer GA, Fukui K Arthroscopic capsule reconstruction in the hip using iliotibial band allograft. Arthrosc Tech 2015; 4: e71–4. 25973378 10.1016/j.eats.2014.11.008 PMC4427639

[52] Chandrasekaran S, Vemula SP, Martin TJ Arthroscopic technique of capsular plication for the treatment of hip instability. Arthrosc Tech 2015; 4: e163–7. 26052494 10.1016/j.eats.2015.01.004 PMC4454834

[53] de Sa D, Horner NS, MacDonald A Arthroscopic surgery for synovial chondromatosis of the hip: a systematic review of rates and predisposing factors for recurrence. Arthrosc J Arthrosc Relat Surg off Publ Arthrosc Assoc N Am Int Arthrosc Assoc 2014; 30: 1499–504.e2. 10.1016/j.arthro.2014.05.033 25064754

[54] Marchie A, Panuncialman I, McCarthy JC. Efficacy of hip arthroscopy in the management of synovial chondromatosis. Am J Sports Med 2011; 39 (Suppl. 1): 126S–31S. 21709042 10.1177/0363546511414014

[55] Byrd JWT, Jones KS, Maiers GP. Two to 10 Years’ follow-up of arthroscopic management of pigmented villonodular synovitis in the hip: a case series. Arthrosc J Arthrosc Relat Surg off Publ Arthrosc Assoc N Am Int Arthrosc Assoc 2013; 29: 1783–7. 10.1016/j.arthro.2013.08.002 24209675

[56] Zhou M, Li Z, Wang Y Arthroscopic debridement and synovium resection for inflammatory hip arthritis. Chin Med Sci J Chung-Kuo Hsueh Ko Hsueh Tsa Chih 2013; 28: 39–43. 10.1016/s1001-9294(13)60017-6 23527805

[57] Bedard NA, Pugely AJ, Duchman KR When hip scopes fail, they do so quickly. J Arthroplasty 2016; 31: 1183–7. 26775065 10.1016/j.arth.2015.12.024

[58] Schairer WW, Nwachukwu BU, McCormick F Use of hip arthroscopy and risk of conversion to total hip arthroplasty: a population-based analysis. Arthrosc J Arthrosc Relat Surg off Publ Arthrosc Assoc N Am Int Arthrosc Assoc 2016; 32: 587–93. 10.1016/j.arthro.2015.10.002 26671201

[59] Conrozier T, Bochu M, Gratacos J Evaluation of the “Lequesnes” false profile’ of the hip in patients with hip osteoarthritis. Osteoarthritis Cartilage 1999; 7: 295–300. 10329304 10.1053/joca.1998.0203

[60] Larson CM, Giveans MR, Taylor M. Does arthroscopic FAI correction improve function with radiographic arthritis? Clin Orthop Relat Res 2011; 469: 1667–76. 21181460 10.1007/s11999-010-1741-6 PMC3094626

[61] Philippon MJ, Briggs KK, Yen Y-M Outcomes following hip arthroscopy for femoroacetabular impingement with associated chondrolabral dysfunction: minimum two-year follow-up. J Bone Joint Surg Br 2009; 91: 16–23. 19091999 10.1302/0301-620X.91B1.21329

[62] Walton NP, Jahromi I, Lewis PL. Chondral degeneration and therapeutic hip arthroscopy. Int Orthop 2004; 28: 354–6. 15597172 10.1007/s00264-004-0585-7 PMC3456896

[63] Kirsch JM, Khan M, Bedi A. Does hip arthroscopy have a role in the treatment of developmental hip dysplasia? J Arthroplasty 2017; 32: S28–31. 28336246 10.1016/j.arth.2017.02.022

[64] Jo S, Lee SH, Wang SI The role of arthroscopy in the dysplastic hip—a systematic review of the intra-articular findings, and the outcomes utilizing hip arthroscopic surgery. J Hip Preserv Surg 2016; 3: 171–80. 27583155 10.1093/jhps/hnv071 PMC5005054

[65] Bogunovic L, Gottlieb M, Pashos G Why do hip arthroscopy procedures fail? Clin Orthop Relat Res 2013; 471: 2523–9. 23637056 10.1007/s11999-013-3015-6 PMC3705062

[66] Parvizi J, Bican O, Bender B Arthroscopy for labral tears in patients with developmental dysplasia of the hip: a cautionary note. J Arthroplasty 2009; 24: 110–3. 10.1016/j.arth.2009.05.021 19596542

[67] Kim C-HO, Dietrich TJ, Zingg PO Arthroscopic hip surgery: frequency of postoperative MR arthrographic findings in asymptomatic and symptomatic patients. Radiology 2017; 283: 779–88. 27930091 10.1148/radiol.2016161078

